# Fibrin clot interference in a human chorionic gonadotrophin assay causing a false Down syndrome screening result

**DOI:** 10.11613/BM.2023.011001

**Published:** 2023-02-15

**Authors:** Arzu Etem Akagac, Hatice Bozkurt Yavuz

**Affiliations:** Department of Clinical Biochemistry, Training and Research Hospital, Usak University, Usak, Turkey

**Keywords:** hCG, prenatal screening, fibrin, preanalytical phase, case report

## Abstract

Serum samples are generally used for the measurement of human chorionic gonadotrophin (hCG) to calculate second-trimester maternal screening results. Lower hCG concentrations correlate with a lower calculated risk of Down syndrome (DS). Hence, erroneously low hCG results due to fibrin clot may lead to misinterpretation. We present a 23-year-old woman with a pregnancy of 17+3 weeks. Blood was taken into the Becton-Dickinson (BD) vacutainer SST-II Advance tube (Ref: 367955). The hCG test was performed on Immulite 2000 XPi analyser (Siemens Healthcare Diagnostics Inc, Tarrytown, USA) with original reagents. The results of the same sample were found as 2566 U/L, 18,153 U/L, and 7748 U/L. Three consecutive results after removal of the small fibrin clot and recentrifugation were 18,878, 20,255, and 22,339 U/L. The risk of DS and MoM for the concentration of 2556 U/L hCG was < 1/10,000 and 0.14, respectively. For a hCG concentration of 20,255 U/L, these values were 1/5632 and 1.13, respectively. Laboratory professionals and technicians should be aware that erroneously low hCG results can be measured with the Immulite 2000 XPi due to interference from small fibrin clots. Falsely underestimated hCG values reduce the MoM values and thus the calculated risk of DS.

## Introduction

Human chorionic gonadotrophin (hCG) is a glycoprotein hormone released by trophoblast cells that is commonly used to diagnose pregnancy, ectopic pregnancy, and hydatiform moles (*1*). The biologically active hCG molecule is formed by the expression of genes encoding both the alpha and beta subunits (*2*). The concentration of hCG is also measured in screening tests in the second trimester along with unconjugated estriol (uE3) and alpha-fetoprotein (AFP). Immunoassay procedures are used to evaluate these tests. In addition, risk is calculated by adding ultrasound findings, patient history, and demographic data (*3*).

Down syndrome (DS) is the most common prenatal chromosomal anomaly, with a prevalence of 1/600 - 1/800 pregnancies (*4*). The second-trimester test has a higher specificity percentage of 80% with a false positive percentage of 5% for DS, and provides useful data to describe various fetal anomalies such as neural tube defects in Western countries (*5*). The goal of second-trimester screening testing is to identify women at increased risk for affected pregnancy and to maximize the achievable benefit for them (*3*).

Blood clotting in the primary tube is a normal process that can also be augmented by using specific clot activators (*6*, *7*). Fibrinogen is a plasma glycoprotein that is converted to fibrin by thrombin during bound proteolysis (*8*, *9*). Fibrinogen is highly heterogenous because of phosphorylation, partial proteolysis or sulfation of amino acids, alternative splicing, and genetic polymorphisms (*10*). Serum separator tubes with separator gel are now generally used in the immunoassay laboratory because the barrier gel that simplifies the rapid and complete separation of serum from the cellular structure of blood (*11*).

As far as we searched, we could not find any study on how the clot in serum affects hCG measurements, although it is known as a source of preanalytical errors. In this case study, we tried to explain how a very small clot in serum, which could not be noticed at the first examination, as a source of preanalytical error, can lead to misinterpretation of the results of DS screening.

## Laboratory analysis

We described here a case of a 23-year-old woman who applied the gynecology policlinic for second-trimester screening in May 2022. This case report was prepared with the informed consent of the patient. The patient had no known history of diabetes mellitus or hypertension. She was a smoker. This was her second pregnancy and she had a healthy living daughter. She did not take any medications and especially anticoagulant therapy. The fetal biparietal diameter (BPD) measured by ultrasonography was 37.3 mm. Blood was taken into the BD vacutainer SST II Advance tube (Ref 367955) in the sampling room of the gynecology and obstetrics department. The sample was centrifuged at 4000 rpm for 10 minutes.

The hCG was measured on an Immulite 2000 (Siemens Healthcare Diagnostics Inc, Tarrytown, USA). It is a solid-phase, two-site chemiluminescent immunometric assay and has two reagents: hCG Reagent Wedge 11.5 mL alkaline phosphatase (REF L2KCG6, lot 469) and hCG Bead Pack coated with monoclonal murine anti-hCG (REF L2CG12, lot 416). The measurement method of hCG was a solid-phase, two-site chemiluminescence immunometric assay. A high concentration of hCG in the sample means a high luminescence signal due to the reaction of antibodies in the reagents. Human chorionic gonadotrophin, AFP and uE3 were measured on the same Immulite 2000 XPi Siemens analyser with a chemiluminescent immunoassay method.

The initial hCG result was 2566 U/L. Alpha-fetoprotein and uE3 concentrations were 45.9 ng/mL and 0.986 ng/mL, respectively, and were measured in duplicate. The results were in agreement with each other for AFP and uE3. Measurement of these three parameters was performed in the clinical chemistry laboratory of a tertiary care hospital.

## Interventions and further investigations

**Figure 1:**
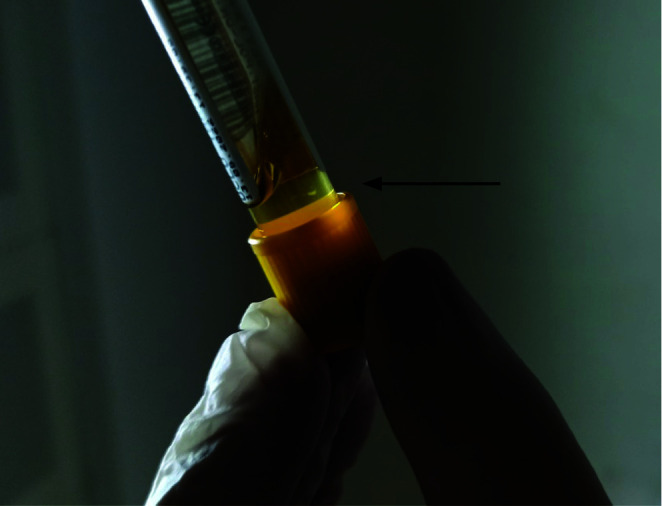
Picture of the fibrin in the serum tube.

When the initial hCG result was obtained as 2566 U/L, it was noted that it was very low compared to the usual results and the test was repeated with the same sample. The repeated hCG result was 18,153 U/L, and there was a significant difference between the two measurements, and the serum was analysed for the third time. The third result was 7748 U/L. After three measurements, the technician informed us and reported the inconsistency of three measurements. The sample showed no haemolysis, lipemia, bilirubinemia, or visible fibrin clots on the first visual examination. The second visual examination of the serum was performed closely under the light by rotating the tube 3-4 times and turning it upside down. The second examination revealed a small fibrin clot that floated in the serum like a thin membrane and it was approximately 2 mm in size (Figure 1). This tiny fibrin clot could not be seen through the tube from a certain angle, but when the serum is moved enough it became visible. Furthermore, this tiny clot was also not visible when the tube was not viewed under the light. After the fibrin clot was removed from the serum and the sample was recentrifuged, the hCG test was performed three times consecutively, and the results were found as 18,878 U/L, 20,255 U/L, and 22,339 U/L.

The Randox International Quality Assessment Scheme maternal screening program was used for the hCG, AFP, and uE3 tests. The performance of all three tests was acceptable in the last six months (< ± 2SDI). Internal quality data also showed no violation of Wetsgard rules in the last six months with an average coefficient of variation of 9.52%, 5.9%, and 12.5% for hCG, AFP, and uE3, respectively.

The manufacturer’s package insert for hCG contained general information about fibrin clots, and it was noted that centrifugation of serum samples without complete clotting can lead to fibrin formation and false results. The package insert did not provide specific explanations about DS screening (*12*).

Manufacturer reported heterophilic antibodies in human serum can react with the immunoglobulins included in the hCG assay components (*12*). However, it is eliminated in this case because repeated hCG measurements with the same sample were significantly different from each other. Additionally, when a fibrin clot was detected, the presence of heterophile antibodies was not considered.

In our hospital, the gynecology and obstetrics department is located in a separate building, and in this building, we have a laboratory unit where the screening samples are accepted and centrifuged. The screening samples are delivered to the laboratory along with the screening request slip, which includes the patient’s ultrasound and demographic data, and medical history. To evaluate the preanalytical process, the time of collection and admission to the laboratory of the serum sample was determined from the Laboratory Information System (LIS), and it was found that the time interval between sample collection and admission was 5 minutes. The patient had requested only a second-trimester screening test as a laboratory test request, so there was no risk of anticoagulant contamination as a result of an error in the sequence of specimen collection. When we asked the technician in charge of the centrifuge, he confided that sometimes they can centrifuge samples as soon as they arrived at the laboratory. Therefore, we thought that in our case the tube might have been centrifuged without sufficient clotting time.

## What happened?

The patient’s blood was centrifuged without waiting long enough and invisible fibrin was formed. The hCG, the first test pipetted by the device probe, was falsely low, although other tests were not affected. If a fibrin clot is detected in laboratory practice, it must be removed from the sample. Then the sample must be centrifuged again at 4000 rpm for 10 minutes and the analysis must be repeated to obtain the correct result.

## Discussion

Hospital laboratories have a tremendous impact on clinical decision-making, with 60-70% of key medication, discharge, and admission decisions being based on clinical laboratory reports (*13*). With this large number of implications, the function of laboratory tests and reports is of absolute importance (*14*). Inappropriate coagulation is the fourth most common (5-10%) cause of specimen-based errors in clinical laboratories after haemolysis, inappropriate specimen volume, and incorrect collection containers (*15*).

In some of our previous studies, we have investigated the effects of fibrin clots in serum on troponin measurements, which is obtained by immunoassay methods such as the hCG test (*16*, *17*). Fibrin clots can lead to erroneous measurements due to insufficient sample volume, sample probe clogging, and assay interference (*18*). In addition, the indicator enzyme can be captured by fibrin, or the reagent antibody can bind nonspecifically to fibrin in the matrix (*19*). In another study, it has been demonstrated that fibrin can be cross-linked with the test antibodies, resulting in a false troponin measurement. Recentrifugation of the sample also reduced the fibrin effect (*20*). It is possible that we obtained three different results in the first serial measurements, because different amounts of fibrin may lead to different reductions in the luminescence signal.

While screening tests are being run on the Immulite 2000, the probe first aspirates serum for the hCG test, and the hCG result is affected by the fibrin clot. For AFP and uE3 tests, we assumed that the clot was not aspirated by the probe because of their pipetting sequence. We assume that the AFP and uE3 tests are therefore not affected by the fibrin clot.

The drop in hCG concentration due to the fibrin clot was particularly dramatic in our case. We calculated the risk for Down syndrome according to the initial and last hCG results. If the hCG result is low, the risk is half as low (1/10,000 - 1/5632). The calculated MoM value is 0.14 at the low hCG result and 1.13 at the last report.

The different hCG results were explained to the technician and other staff. The effects of early centrifugation of the sample on the patient’s results were demonstrated. Necessary precautions were taken for the samples to be kept in the laboratory for at least 30 minutes and then centrifuged. Our case highlights the importance of appropriate sample preparation and the impact of poor sample integrity on clinical laboratory results. A false positive result on second-trimester screening due to preanalytical interference may lead to unnecessary amniocentesis, and anxiety in the family. A false negative result can also lead to the underdiagnosis of DS. Both situations may cause irreversible results.

## What you can do in your laboratory to prevent such errors

The presence of fibrin in the sample, as with other tests, affects hCG results and can lead to erroneous results in maternity screening. Care should be taken with extreme torque values.
Fibrin is not easily detected at first glance, and specimens should be examined carefully, especially under light.
The preanalytical phase within the laboratory and the procedures should be carefully followed.
Laboratory technicians should be informed of preanalytical problems associated with fibrin clots.

## References

[1] Ding X Yang KL. Antibody-Free Detection of Human Chorionic Gonadotropin by Use of Liquid Crystals. Anal Chem. 2013;85:10710–6. 10.1021/ac400732n 24147645

[2] Boothby M Kukowska J Boime I. Imbalanced synthesis of human choriogonadotropin alpha and beta subunits reflects the steady-state levels of the corresponding mRNAs. J Biol Chem. 1983;258:9250–3. 10.1016/S0021-9258(17)44659-X 6192128

[3] Xie Z Lu S Li H. Contingent triple-screening for Down’s syndrome in the second trimester: a feasibility study in Mainland Chinese population. Prenat Diagn. 2010;30:74–6. 10.1002/pd.2412 19967750

[4] Snijders RJ Sundberg K Holzgreve W Henry G Nicolaides KH. Maternal ageand gestation-specific risk for trisomy 21. Ultrasound Obstet Gynecol. 1999;13:167–70. 10.1046/j.1469-0705.1999.13030167.x 10204206

[5] Driscoll DA Gross SJ. Professional practice guidelines committee. Screening for fetal aneuploidy and neural tube defects. Genet Med. 2009;11:818–21. 10.1097/GIM.0b013e3181bb267b 19915395 PMC3111043

[6] Dimeski G Johnston J Masci PP Zhao KN Brown N. Evaluation of the Greiner Bio-One serum separator BCA Fast Clot tube. Clin Chem Lab Med. 2017;55:1135–41. 10.1515/cclm-2016-0806 28076307

[7] Cadamuro J Mrazek C Leichtle AB Kipman U Felder TK Wiedemann H Influence of centrifugation conditions on the results of 77 routine clinical chemistry analytes using standard vacuum blood collection tubes and the new BD-Barricor tubes. Biochem Med (Zagreb). 2018;28:010704. 10.11613/BM.2018.010704 29187797 PMC5701775

[8] Pratt KP Côté HC Chung DW Stenkamp RE Davie EW. The primary fibrin polymerization pocket: three-dimensional structure of a 30-kDa C-terminal gamma chain fragment complexed with the peptide Gly-Pro-Arg-Pro. Proc Natl Acad Sci USA. 1997;94:7176–81. 10.1073/pnas.94.14.7176 9207064 PMC23783

[9] Yang Z Mochalkin I Doolittle RF. A model of fibrin formation based on crystal structures of fibrinogen and fibrin fragments complexed with synthetic peptides. Proc Natl Acad Sci USA. 2000;97:14156–61. 10.1073/pnas.97.26.14156 11121023 PMC18887

[10] Henschen-Edman AH. Fibrinogen non-inherited heterogeneity and its relationship to function in health and disease. Ann N Y Acad Sci. 2001;936:580–93. 10.1111/j.1749-6632.2001.tb03546.x 11460517

[11] Bush VJ Janu MR Bathur F Wells A Dasgupta A. Comparison of BD Vacutainer SST Plus Tubes with BD SST II Plus Tubes for common analytes. Clin Chim Acta. 2001;306:139–43. 10.1016/S0009-8981(01)00396-5 11282105

[12] Immulite 2000 hCG Siemens Reagent Insert. Available from: https://www.uk-essen.de/zentrallabor/neu/zenlabhp/media/files/HCG_Immulite_Packungsbeilage.pdf. Accessed September 16th, 2022.

[13] Forsman RW. Why is the laboratory an afterthought for managed care organizations? Clin Chem. 1996;42:813–6. 10.1093/clinchem/42.5.813 8653920

[14] Plebani M. Charting the course of medical laboratories in a changing environment. Clin Chim Acta. 2002;319:87–100. 10.1016/S0009-8981(02)00028-1 11955484

[15] Lippi G Myer AV Cadamuro J Simundic AM. Blood Sample Quality. Diagnosis (Berl). 2019;6:25–31. 10.1515/dx-2018-0018 29794250

[16] Yavuz HB. Erroneously high troponin measurement caused by fibrin clot: Two cases. Int J Med Biochem. 2021;4:208–10. 10.14744/ijmb.2021.64872

[17] Etem AA. False High Troponin Level Originated From Fibrin in Serum: Two Cases. Turk Klin Biyokim Derg. 2020;18:42–5.

[18] Sharif W Stretton A Cornes M Shetty C. Frequency of latent fibrin in centrifuged serum specimens. Clin Chem Lab Med. 2019;57:eA1–87.33577727

[19] Nosanchuk JS. Falseincreases of troponin I attributed to incomplete separation of serum. Clin Chem. 1999;45:714. 10.1093/clinchem/45.5.714 10222366

[20] Dimeski G. Evidence on the cause of false positive troponin I results with the Beckman AccuTnI method. Clin Chem Lab Med. 2011;49:1079–80. 10.1515/CCLM.2011.163 21428855

